# Noise-Aware Explanatory Enrichment of Origin–Destination Frequency Tensors for Interpretable Mobility Clustering

**DOI:** 10.3390/s26216999

**Published:** 2026-11-05

**Authors:** Dani Marchuet, Javi Palanca, Vicent Botti

**Affiliations:** 1 Valencian Research Institute for Artificial Intelligence (VRAIN), Universitat Politècnica De València, Camí De Vera s/n, 46022 Valencia, Spain; jpalanca@dsic.upv.es (J.P.); vbotti@dsic.upv.es (V.B.); 2 Valencian Graduate School and Research Network of Artificial Intelligence (ValgrAI), Camí de Vera s/n, 46022 Valencia, Spain

**Keywords:** mobility analytics, origin–destination tensor, HDBSCAN, BIRCH, explainable clustering, partition stability, mobility modelling

## Abstract

This paper proposes a noise-aware, iterative methodology for enriching an initial mobility clustering through the sequential addition of explanatory variables. The framework separates one-off density-based cluster discovery from scalable characterisation over a fixed analytical population. Candidate variables are retained only when they improve the predefined distributional criteria and preserve continuity with the latest accepted partition. Evaluation on a large sensing-based mobility dataset from Valencia shows that the proposed architecture supports scalable and auditable explanatory enrichment while keeping the observation set unchanged and explicitly separating distributional admissibility from inter-partition continuity.

## 1. Introduction

Urban mobility analysis supports transport planning, congestion management and service design by identifying recurrent flows between locations. Privacy-preserving origin–destination representations are particularly valuable because they retain aggregate spatio-temporal structure without requiring directly identifiable trajectories.

Origin–destination frequency tensors provide a structured representation of temporal and spatial mobility interactions. Earlier work introduced wavelet-based tensors for mobility-pattern extraction [1,2], followed by an incremental explanatory variable enrichment methodology that progressively incorporated explanatory attributes and assessed their effect on clustering [3].

This study extends the work of [3] through three additions: one-off HDBSCAN discovery of a density-supported analytical population; scalable characterisation through a combined BIRCH–MiniBatchKMeans procedure in which the global stage operates on CF-tree leaf summaries; and a reproducible two-stage rule separating distributional admissibility from structural continuity.

The previous framework left three limitations: noisy observations remained in every enrichment stage, HDBSCAN was repeatedly executed on millions of records, and the continued use of density-based clustering hindered automation across many candidate variables [3,4,5,6].

The proposed framework separates discovery from characterisation. HDBSCAN is executed once to identify density-supported observations and the reference resolution. For every subsequent representation, BIRCH compresses the fixed retained population into clustering-feature summaries, and MiniBatchKMeans performs global characterisation over the resulting leaf summaries rather than over all original observations. Candidates are accepted only when they improve both distributional criteria and continuity with the latest accepted partition.

The principal contributions are as follows: (i) separation of density-based discovery from scalable characterisation; (ii) a combined BIRCH–MiniBatchKMeans architecture that hands compressed CF-tree leaf summaries to the global centroid stage and reduces full-scale runtime relative to HDBSCAN under the controlled benchmark; (iii) a fixed observation mask and requested resolution for controlled inter-epoch comparison; and (iv) an entropy–Gini–Simpson–NMI rule governing cumulative enrichment. Section 2 reviews the essential background; Section 3 defines the method; Section 4 and Section 5 present and discuss the results; and Section 6 concludes the study.

## 2. Related Work

Figure 1 links the sensing and ingestion layer to tensor construction and the analytical pipeline. Section 2 therefore focuses on four elements needed to position the contribution: privacy-preserving acquisition, origin–destination tensor modelling, explainable clustering and partition assessment.

### 2.1. Sensing Infrastructure and Data Acquisition

**Dataset and anonymisation.** The case study comprises approximately 17.7 million mobile-device detections recorded during May 2025 at 136 sensing locations in Valencia. Detected MAC addresses were irreversibly salted and hashed at the sensing point; raw identifiers were not transmitted. A common salt kept pseudonymous identifiers stable across sensors during the one-month observation window, permitting aggregated origin–destination reconstruction while bounding longitudinal traceability [1,2].

**Smart-city communication.** Sensors transmitted pseudonymous observations through LoRaWAN and MQTT or through HTTPS and FIWARE-compatible interfaces [7,8,9]. This architecture separates local detection and pseudonymisation from transport, payload decoding and context storage.

**Passive sensing and measurement uncertainty.** The sensing infrastructure can passively scan broadcast Wi-Fi, Bluetooth and Bluetooth Low Energy activity without establishing a connection to the observed device; however, the present case study uses only detections derived from broadcast Wi-Fi frames. Within a configurable detection window, repeated Wi-Fi observations are deduplicated by pseudonymous identifier, time-stamped at window closure, buffered locally and transmitted as compact counter and identifier payloads. Detection is therefore an observation of active Wi-Fi emissions rather than a direct count of people or complete device trajectories. Its precision depends on Wi-Fi activation, scan duration, receiver sensitivity, radio propagation and occlusion, device transmission behaviour, identifier randomisation, and the temporal alignment of detections across locations. Communication capacity and buffering may delay delivery, although stored time-stamps preserve the acquisition window; interruptions, incomplete coverage or inconsistent Wi-Fi detectability can still fragment an apparent journey. These characteristics motivate treating low-support transitions as analytically uncertain rather than automatically erroneous.

**Transition reconstruction.** Temporally ordered detections of the same pseudonymous identifier at different sensors define candidate transitions. These are aggregated by origin, destination and temporal interval to construct frequency series and tensor observations; clustering uses aggregated data rather than raw identifiers or directly identifying trajectories [1,2].

**Spatial coverage.** Observed mobility is bounded by the 136 registered sensing locations: areas without functioning detectors cannot generate origins or destinations, and local radio visibility, crowd density, device discoverability and communications availability affect the recorded volume. Because validated sensing ranges and device-level error rates were unavailable, the analysis is interpreted as mobility observed by this infrastructure rather than as a complete census.

**Sources of uncertainty.** Missed or duplicate detections, temporal batching, location association and communication loss can remove, inflate or reorder transitions. MQTT delivery also depends on the configured quality of service [8]. Aggregation reduces isolated effects but not systematic coverage or transmission bias; quantitative propagation remains future work because validated sensor-level error rates were unavailable.

### 2.2. Origin–Destination Tensor Modelling

Conventional origin–destination matrices summarise flows between spatial units but provide limited multi-scale temporal information. The wavelet frequency tensor transformed origin–destination frequency series into a representation suitable for privacy-preserving pattern extraction [1], and the later origin–destination frequency tensor generalised this structure to discrete systems with states, transitions and temporally varying frequencies [2].

The present study preserves the tensor as the structural mobility representation and adds external attributes only to characterise the resulting partitions. Latitude, longitude, journey duration, temporal period and route distance therefore complement rather than redefine the underlying transition model. At the application level, origin–destination demand has also been analysed jointly with contextual information on nearby services, public-transport supply, multiple operators and mobility indicators to support scenario comparison and identify service–demand mismatches [10]. This illustrates the practical value of enriching origin–destination representations with external context, although the present contribution differs by evaluating candidate attributes sequentially within an unsupervised partition-enrichment framework. Related spatio-temporal tensor research also supports tensor co-clustering for identifying persistent mobility systems [11].

### 2.3. Explainable Clustering

Clustering lacks labelled targets, so interpretability must consider transparency, fidelity and comprehensibility as well as partition quality [12]. Existing methods are either intrinsically interpretable or provide post hoc explanations, but post hoc surrogates may not faithfully represent how the original groups were formed.

The enrichment framework in [3] instead incorporated semantically meaningful variables into the clustering space and measured their effect on the partition. The present work retains that principle but assigns different roles to the algorithms: HDBSCAN performs one-off density discovery and noise identification, while the combined BIRCH–MiniBatchKMeans procedure performs repeated characterisation over the fixed retained population [4,5,6,13,14,15,16].

### 2.4. Partition Stability Assessment

Partition assessment must distinguish the quality of one solution from agreement between solutions [17,18,19]. Silhouette, Davies–Bouldin and Calinski–Harabasz evaluate cohesion and separation and are used for the initial HDBSCAN discovery and BIRCH baseline [20,21,22].

For enrichment, relative Shannon entropy and normalised Gini–Simpson diversity describe membership distribution [23,24], while NMI provides a label-invariant measure of shared information between consecutive accepted partitions [25,26,27]. ARI may be reported diagnostically but is not used in the acceptance rule because pairwise agreement can decrease under refinement or fragmentation [28].

The proposed rule combines these roles sequentially. A candidate first must improve both distributional indicators relative to the latest accepted epoch. Only then is NMI computed, and the candidate is accepted if continuity exceeds that of the preceding accepted transition. Rejected candidates do not alter the accepted feature representation or reference partition.

## 3. Materials and Methods

The method operates on a tensor-derived feature matrix in which each row represents one observation and the columns encode the selected structural coefficients. Figure 2 summarises the two-stage accept–reject rule, while Figure 3 presents its sequential application across candidate epochs. Candidate variables are evaluated sequentially: rejected candidates do not enter the cumulative feature space, whereas each accepted partition becomes the reference for the next comparison.

The procedure separates one-off density-based discovery from repeated characterisation. The discovery stage defines a density-supported analytical population and a reference resolution. A scalable characterisation algorithm then establishes the operational baseline and evaluates an application-defined sequence of candidate variables. Each accepted candidate updates the cumulative representation and reference partition; each rejected candidate leaves both unchanged.

Section 3.1 and Section 3.2 define root-partition discovery and construction of the fixed analytical population. Section 3.3 and Section 3.4 define combined BIRCH–MiniBatchKMeans characterisation and its baseline. Section 3.5 and Section 3.6 formalise iterative enrichment and the two-stage evaluation used to accept or reject each candidate variable. The case-specific implementation and empirical sequence are reported separately in Section 4.

### 3.1. HDBSCAN Root-Partition Discovery

Let *X*_0_ denote the matrix containing only the structural tensor features. Before density-based discovery, continuous features are standardised by subtracting their mean and dividing by their standard deviation; zero-variance columns are removed to avoid undefined scaling and non-informative dimensions. No candidate explanatory variables are included at this stage.

HDBSCAN is applied once to *X*_0_ because it can identify irregular groups with heterogeneous densities without requiring their number in advance [4,5,6]. Its hyperparameters are selected for the application and reported with the experiment. The condensed hierarchy identifies persistent components, while observations unsupported by stable density regions receive the noise label.

The resulting label vector is denoted by *y*^H^, where *y*^H^_i_ = −1 identifies noise and non-negative values identify density-supported groups. This preliminary HDBSCAN phase is designed to recover a robust root partition from the structural tensor features before any explanatory variable is introduced. The complete output is treated as the discovery reference rather than as ground truth: it defines the valid observation set *V* and the reference resolution *K* used to configure the subsequent BIRCH characterisation stage.

Candidate discovery configurations are evaluated jointly through Silhouette, Davies–Bouldin and Calinski–Harabasz indices [20,21,22]. Higher Silhouette and Calinski–Harabasz values and lower Davies–Bouldin values indicate more favourable geometry. These metrics are computed only for density-supported groups, while the excluded proportion is reported separately. The selected configuration is executed once and returns the reference labels, validity mask and reference resolution *K*.

### 3.2. Noise Elimination

The validity mask removes observations labelled −1 and constructs the fixed analytical matrix *X*_V_. This same mask is applied to every candidate variable, preserving observation identity and order across epochs. Consequently, later comparisons are not confounded by changes in sample composition.

Fixing the analytical population improves comparability and reduces the records entering repeated preprocessing and CF-tree construction. It does not imply that excluded observations are erroneous. In the present Wi-Fi-based, passive and non-connected sensing case, an observation can be weakly supported because detections depend on Wi-Fi activity, device discoverability, radio visibility, the configured scanning window and sensitivity, and successful communication and ingestion. Privacy mechanisms that rotate Wi-Fi identifiers may also fragment the apparent continuity of a physical device. The HDBSCAN noise set may therefore combine acquisition artefacts with rare, spatially isolated, temporally sparse or genuinely non-recurrent mobility. The retained and excluded counts, computational effect and substantive scope of inference must consequently be reported for each application.

### 3.3. Justification of Combined BIRCH–MiniBatchKMeans Characterisation

The repeated characterisation stage is implemented as a combined BIRCH–MiniBatchKMeans procedure rather than as either algorithm in isolation. BIRCH first compresses the observations into a CF-tree whose leaf subclusters retain sufficient statistics. MiniBatchKMeans then performs the requested global partitioning over the leaf-subcluster centroids, with the associated subcluster weights, instead of revisiting every original observation during the global stage. HDBSCAN and this combined procedure retain distinct geometric roles: HDBSCAN discovers irregular groups across heterogeneous density regions without requiring a predefined number of clusters, whereas the global centroid-based stage applied to the CF-tree summaries favours compact, variance-minimising partitions. Setting its parameter to the reference value *K* keeps the requested resolution comparable across epochs; it does not imply equivalent cluster shapes or memberships.

Scalability and methodological fit guided algorithm selection. Spectral and conventional agglomerative approaches require graph or distance structures that are prohibitive for millions of records, while K-Means and Gaussian mixtures impose stronger shape assumptions [29,30]. In the proposed pipeline, BIRCH converts the retained observations into clustering features containing counts, linear sums and squared sums, and the subsequent MiniBatchKMeans stage receives those summaries rather than the complete observation matrix [13]. This compressed hand-off is the methodological mechanism that makes repeated global characterisation across enriched feature representations computationally tractable while exposing intermediate compression diagnostics. A standalone MiniBatchKMeans fit on the original matrix is retained only as an ablation control: it measures the cost and occupancy of bypassing the CF-tree, but it does not reproduce the proposed summarisation-and-hand-off architecture.

Table 1 summarises why the components perform complementary rather than interchangeable roles. HDBSCAN is retained for one-off density discovery and noise identification. During repeated characterisation, BIRCH supplies the compressed CF-tree representation and MiniBatchKMeans supplies the global centroid partition over its leaf summaries; together they constitute the scalable characterisation procedure evaluated in Section 4.

At epoch *e*, BIRCH receives a matrix *X*^(*e*)^ defined over the fixed population *V*. Continuous features present in that matrix are standardised to zero mean and unit variance using statistics fitted on *V*; zero-variance columns are removed. Categorical candidates must be encoded without imposing unsupported ordinality, and their encoding and scaling treatment must be stated explicitly. The BIRCH threshold, branching factor, global clustering estimator and reproducibility settings remain fixed across candidate epochs and are reported with the experimental implementation.

The global characterisation stage requests the reference resolution *K*, although the number of labels receiving observations, *K*_occ_, may be lower. Effective occupancy and centroid utilisation are therefore reported separately and do not modify the requested resolution or the acceptance rule.

### 3.4. Baseline Characterisation

Before adding explanatory variables, the combined BIRCH–MiniBatchKMeans procedure is fitted to *X*_V_ to obtain the tensor-only baseline partition *y*^(0)^. BIRCH first constructs the CF-tree summaries, and MiniBatchKMeans then forms the requested global partition from the leaf-subcluster centroids and weights. The restricted discovery partition *y*_V_^H^ and *y*^(0)^ are compared once over the same observations to assess the algorithmic hand-off from density discovery to scalable characterisation. This one-off comparison evaluates whether the combined baseline preserves sufficient information from the robust HDBSCAN root structure, but it neither initialises an acceptance threshold nor forms part of the enrichment chain. The iterative rule begins only with the first candidate transition from Epoch 0 to candidate Epoch 1; thereafter, each candidate is compared with the most recent accepted partition produced by the combined procedure.

### 3.5. Multi-Epoch Enrichment

Let *C* = (*c*_1_, …, *c*_m_) be an application-defined ordered set of candidate variables. For candidate *c*_e_, the method appends its encoded features to the matrix associated with the latest accepted epoch, applies the fixed preprocessing and characterisation configuration, and obtains candidate partition *y*^(*e*)^. Acceptance updates the cumulative matrix and reference partition; rejection discards the candidate and preserves the latest accepted state. The process continues until the ordered candidate set is exhausted or an application-defined stopping condition is reached. Figure 2 defines the decision rule and Figure 3 shows its generic sequential application, while the case-specific candidate sequence is reported in Section 4.

### 3.6. Partition Stability Evaluation

The decision procedure in Figure 2 contains two sequential gates. Stage 1 evaluates the candidate partition under predefined distributional criteria for the invariant observation set. Stage 2 evaluates continuity with the latest accepted partition. Separating these roles prevents high inter-partition agreement from compensating for a candidate that fails the internal criteria and prevents an internally improved but structurally unrelated partition from entering the accepted chain.

**Stage 1—Internal validation of the candidate partition.** Let *n*_k_ be the membership count of label *k*, *N* the number of retained observations and *p*_k_ = *n*_k_/*N*. Relative Shannon entropy and normalised Gini–Simpson diversity evaluate the complete membership distribution against the fixed reference resolution *K*. A candidate partition passes this mandatory gate only if both indicators improve relative to the latest accepted partition *a*(*e*); otherwise, it is rejected and Stage 2 is not executed. This is an operational criterion of the framework rather than a universal preference for balanced partitions.

Using the fixed requested resolution *K*, relative Shannon entropy is defined as *H*_rel_ = −[Σ*_k_*_=1_*^K^ p_k_* log *p_k_*]/log *K*, with the convention 0 log 0 = 0. Normalised Gini–Simpson diversity is defined as *D*_GS_ = [1 − Σ*_k_*_=1_*^K^ p_k_*^2^]/[1 − 1/*K*]. Both measures therefore lie in [0, 1] and use the invariant requested resolution *K*, rather than the epoch-specific occupied count, as their normalisation reference.

Stage 1 determines admissibility under the predefined distributional criteria. Its role is not to establish causal relevance, but to require a measurable and reproducible improvement before continuity is assessed. The empirical interpretation of accepted and rejected candidates belongs to the application results rather than to the general method.

**Stage 2—Comparison with the latest accepted BIRCH partition.** A candidate that passes Stage 1 is compared with *y*^(*a*(*e*))^ by computing NMI. Because cluster identifiers are arbitrary, NMI measures shared information without requiring label correspondence. For the first candidate, the reference is the BIRCH tensor baseline *y*^(0)^; for every later candidate, the reference is the latest accepted BIRCH epoch. The preliminary HDBSCAN-to-Epoch-0 NMI is not part of this sequence and does not initialise the acceptance rule. Let *q_e_* = NMI(*y*^(*a*(*e*))^, *y*^(*e*)^) denote the continuity value of an eligible candidate transition. For Epoch 1, *q*_1_ is accepted as the initial value of the BIRCH continuity sequence after Stage 1 has been passed. For every subsequent eligible candidate *e* > 1, acceptance requires *q_e_* ≥ *q_r_*, where *q_r_* is the NMI of the most recent accepted BIRCH transition. Equality therefore satisfies the rule. If the inequality fails, the previous accepted state and its continuity reference are retained. Rejected candidates never enter the cumulative feature space.

For two partitions *U* and *V*, NMI uses arithmetic-mean normalisation: NMI(*U*, *V*) = 2*I*(*U*; *V*)/[*H*(*U*) + *H*(*V*)], where *I*(*U*; *V*) = Σ*_i_*_,*j*_ *p_ij_* log[*p_ij_*/(*p_i_p_j_*)] and *H*(*U*) = −Σ*_i_ p_i_* log *p_i_*, with *H*(*V*) defined analogously. The same logarithm base is used throughout, so its choice does not affect the normalised value.

NMI is therefore restricted to structural continuity and is not interpreted as evidence that a candidate is intrinsically informative. The two stages answer different questions: Stage 1 tests admissibility under the distributional criteria, whereas Stage 2 tests preservation of accumulated partition information.

## 4. Results

Figure 4 instantiates the generic method of Section 3 for the Valencia case study. The experiment aggregates approximately 17.7 million detections into 5,897,113 tensor observations. HDBSCAN defines the retained analytical population and reference resolution; the combined BIRCH–MiniBatchKMeans procedure then establishes the tensor-only baseline and evaluates the ordered explanatory candidates. Table 2 reports the baseline comparison, Table 3 the controlled runtime benchmark and full-scale standalone MiniBatchKMeans ablation, Table 4 the performance profile across cumulative representations, Table 5 the preliminary shuffled-control assessment, and Table 6 the resulting acceptance sequence.

### 4.1. Case Study and Accepted Enrichment Sequence

All runs use the same 1,763,591 retained observations, row order and requested resolution of *K* = 2176. The HDBSCAN discovery configuration is *min_cluster_size* = 100, *min_samples* = 16 and *cluster_selection_epsilon* = 0.1. Continuous features are standardised independently at each candidate epoch using statistics fitted on the full retained population, and zero-variance columns are removed.

The BIRCH implementation uses threshold 0.1 and branching factor 50. Its leaf-subcluster centroids are supplied to MiniBatchKMeans with 2176 requested global centroids, batch size 5000, random state 42 and three initialisations [15,16,31]. This configuration remains unchanged throughout the enrichment sequence.

The ordered candidates are origin and destination coordinates, journey duration, temporal representation and route distance. The initial numerical hour representation is tested and rejected before the categorical temporal alternative is evaluated. The accepted categorical representation uses three mutually exclusive unscaled indicators: night (21:00–06:00), morning (06:00–12:00) and afternoon/evening (12:00–21:00). Route distance denotes travel through the transport network rather than straight-line separation. This experimental sequence is depicted in Figure 4 and evaluated in Section 4.5 and Section 4.7, with Section 4.6 providing the preliminary control.

The accepted representation extends two successive stages of the earlier work. The origin–destination frequency tensor in Ref. [2] defined the structural baseline through tensor cuts that encode accumulated transition frequencies. The enrichment study in Ref. [3] then added and accepted origin and destination latitude–longitude variables. The present framework retains that spatial enrichment and further accepts journey duration, categorical day period and route distance; numerical hour is explicitly rejected and reformulated before the categorical alternative is accepted. The comparison therefore shows continuity with Ref. [3] while extending the accepted representation and making rejection and reformulation auditable.

### 4.2. Root Cluster and Baseline Validation

HDBSCAN retained 1,763,591 density-supported observations and established 2176 valid clusters, thereby fixing the analytical population and requested characterisation resolution before enrichment. As reported in Table 2, the one-off comparison between the restricted HDBSCAN root partition and the combined BIRCH–MiniBatchKMeans Epoch 0 baseline yields NMI = 0.768 and ARI = 0.531. These values assess the algorithmic hand-off: they indicate substantial shared information but only moderate pairwise correspondence, not geometric equivalence. Silhouette decreases from 0.835 to 0.276 and Davies–Bouldin worsens from 0.628 to 1.198, showing that the combined baseline is comparatively weaker under cohesion-and-separation criteria. Conversely, Calinski–Harabasz rises from 24,187.035 to 6,920,681.897; this index is closely related to the between-/within-cluster dispersion criterion favoured by centroid-based variance minimisation and therefore strongly favours the MiniBatchKMeans-type global geometry. The large CH increase should not override the less favourable Silhouette and Davies–Bouldin evidence. Epoch 0 is adopted as the operational starting point for the combined enrichment chain because it is constructed over the HDBSCAN-defined population and resolution. The value 0.768 validates this hand-off but does not initialise the acceptance threshold; the first eligible acceptance comparison is Epoch 0 versus candidate Epoch 1.

### 4.3. Controlled Runtime Benchmark

A controlled single-run benchmark compared HDBSCAN with the complete proposed BIRCH–MiniBatchKMeans procedure on samples of 250,000, 500,000, 1,000,000 and 1,763,591 observations within a shared Slurm-managed HPC environment (AMD EPYC 7453 processor, 56 logical CPUs, 503.7 GB installed RAM, Python 3.8.19 and scikit-learn 1.7.0). These specifications describe node capacity, not exclusive job resources; concurrent workload was not controlled. In the combined procedure, BIRCH first constructs the CF-tree and MiniBatchKMeans then performs global clustering over the resulting leaf-subcluster centroids rather than directly over all observations. Table 3 and Figure 5 show a consistent runtime advantage for this procedure over HDBSCAN across the tested range: at full scale, it required 4440.321 s (74.005 min), whereas HDBSCAN required 16,879.325 s (281.322 min), corresponding to a 3.80× speed-up and an approximately 73.7% runtime reduction. This measured reduction supports the proposed computational architecture as a whole. The 74.005 min value is the runtime measured in this dedicated benchmark, not an upper bound or an estimate of intrinsic CF-tree cost; it is interpreted separately from the reproduction profiled in Section 4.4. A standalone MiniBatchKMeans ablation was additionally fitted to the same standardised 15-feature tensor matrix at the full retained population, using 2176 requested centroids, batch size 5000, random state 42 and three initialisations. It required 33.112 s (0.552 min), reached a peak RSS of 0.619 GB and occupied 1266 centroids (58.180%), compared with complete occupancy for the combined-procedure baseline. This ablation confirms that the centroid update itself is inexpensive when run directly on the tensor matrix; it does not reproduce the proposed CF-tree summarisation and compressed hand-off, nor does it test repeated enriched representations. Because the benchmark and ablation differ in execution scope and output, no standalone-to-CF-tree overhead ratio is inferred. Each configuration was measured once, and peak memory was recorded only for the standalone ablation and the separate representation-level profile.

### 4.4. Performance Profile Across Combined Characterisation Epochs

This profile complements the dedicated benchmark in Section 4.3 by examining computational behaviour across cumulative feature representations. Its tensor-only baseline required 1.983 min, whereas the separate full-scale benchmark reported 74.005 min. Conclusions are drawn within each experiment, and their absolute runtimes are not combined to estimate CF-tree overhead. Both were run in a shared Slurm-managed HPC environment, so elapsed times are specific to their execution conditions.

The complete BIRCH–MiniBatchKMeans sequence was profiled on the fixed set of 1,763,591 observations using a node with an AMD EPYC 7453 processor, 56 logical CPUs and 503.7 GB installed RAM; these figures describe node capacity, not job-exclusive resources. The procedure used threshold 0.1, branching factor 50 and MiniBatchKMeans with 2176 requested centroids, batch size 5000, random state 42 and three initialisations. Table 4 and Figure 6 report only computational measurements for each evaluated representation: feature dimensionality, runtime, peak resident-set size and CF-tree size. No validation metric or acceptance outcome is used in this performance analysis.

The tensor-only baseline required 1.983 min and 1.334 GB peak RSS. Adding spatial coordinates increased the profile to 4.020 min and 2.151 GB, while the duration representation required 5.005 min and 2.341 GB. The rejected candidate (numerical hour) is not included in the profile because it does not form part of the cumulative chain. The largest increase occurred for the categorical day-period representation, which required 110.186 min and 3.712 GB; the route-distance representation required 111.263 min and 3.843 GB. The increase from approximately 5 to 110 min after introducing the three unscaled binary day-period indicators is consistent with the 0.1 threshold reducing CF-tree compression for discrete 0/1 transitions and with the substantial expansion of the tree, although this remains a plausible interpretation rather than a demonstrated causal mechanism. Over the same sequence, CF-tree leaf nodes increased from 3519 to 28,932 and leaf subclusters from 105,709 to 857,769. These measurements show that performance depends on the cumulative feature representation and the resulting CF-tree complexity, even when sample size, BIRCH parameters and requested resolution remain fixed.

### 4.5. Internal Validation of Candidate Variables

The Stage 1 values confirm the successive internal decisions. Epoch 1 increases relative entropy from 0.528 to 0.680 and Gini–Simpson diversity from 0.908 to 0.977. Epoch 2 increases them to 0.686 and 0.978. The numerical hour-of-day candidate then reduces both measures to 0.564 and 0.918 and is rejected. Against the unchanged Epoch 2 reference, the categorical day-period candidate increases the indicators to 0.899 and 0.998; route distance subsequently raises them to 0.920 and 0.999. Thus, all candidates except numerical hour of day pass Stage 1 and become eligible for NMI comparison.

Previous research shows that irrelevant or noisy dimensions can modify the geometry recovered by clustering algorithms and affect the behaviour of internal validity indices, although the magnitude of this effect depends on the noise distribution, data dimensionality, scaling procedure, clustering method and evaluation metric [32,33]. In particular, Ref. [32] analyses irrelevant features for K-Means across several clustering metrics, whereas Ref. [33] compares the response of cluster-validity indices under outliers and noisy dimensions using multiple clustering algorithms. These studies motivate caution rather than a universal claim that every non-informative variable must degrade every quality measure. Accordingly, Stage 1 imposes an empirical requirement: a candidate representation must improve both relative entropy and normalised Gini–Simpson diversity with respect to the latest accepted partition before structural continuity is assessed. This interpretation is also consistent with the authors’ previous HDBSCAN-based enrichment study, in which origin and destination latitude–longitude variables were incorporated and evaluated through explicit cluster-quality and partition-comparison measures rather than accepted solely on the basis of their semantic relevance [3]. The present results reinforce this rationale: numerical hour of day fails the distributional-admissibility gate, whereas its categorical day-period reformulation improves both indicators. Stage 1 thus operates as a data-dependent filter that rejects candidate representations that do not improve the predefined distributional criteria.

### 4.6. Preliminary Shuffled-Control Assessment

The root partition represents structural mobility frequencies before any additional characterising attribute is introduced. Its tensor-only BIRCH–MiniBatchKMeans counterpart establishes Epoch 0 as the operational baseline, after which Stage 1 evaluates the incremental contribution of each candidate against the latest accepted partition using relative entropy and Gini–Simpson diversity. A separate preliminary control assessed the first two enrichment candidates and five independently shuffled variants during an earlier phase of the research. This control used the first half of May 2025 and a different candidate order; its epoch labels and values are therefore not directly comparable with Section 4.5 and Table 6. The rows labelled “Real” in Table 5 denote the unshuffled candidate representations. Shuffling preserves each variable’s marginal distribution while breaking its association with the tensor observations. Candidate–reference improvement and real–shuffled comparison thus address different questions: the former evaluates distributional contribution under the methodological rule, whereas the latter examines whether favourable indicator values also arise after random reassignment.

The added variables make a measurable incremental contribution in terms of the predefined distributional criteria. In the main experiment, coordinates increase relative entropy from 0.528 to 0.680 and Gini–Simpson diversity from 0.908 to 0.977; duration then produces smaller but positive increases to 0.686 and 0.978, respectively. Within the separate preliminary reproduction reported in Table 5, adding duration to the coordinate-enriched representation likewise increases relative entropy from 0.7651 to 0.7911 and Gini–Simpson diversity from 0.9908 to 0.9935. These comparisons support Stage 1 validation relative to the preceding accepted representation. The methodological contribution is the computable, sequential evaluation of such increments within an enrichment process that can be automated; it is distinct from statistical significance, for which no formal test is reported. For both candidates in Table 5, the real partition occupies all 2176 requested clusters, while at least one shuffled run leaves one or two unoccupied. The supported enrichment claim concerns the observed increments along the unshuffled candidate sequence relative to its preceding accepted representations, not whether individual real values exceed the shuffled ranges. The overlap shows that similar distributional values can also arise after random reassignment across the five shuffle seeds; it does not negate the observed real-sequence increments, but prevents interpreting them alone as evidence of attribute-specific semantic relevance or superiority over random reassignment. These seeds vary the reassignment of candidate values, not the clustering initialisation or candidate order, and therefore do not demonstrate seed-wise stability of the real acceptance sequence. NMI serves only the separate Stage 2 purpose of assessing structural continuity with the latest accepted partition. The main acceptance sequence remains unchanged, and the preliminary control is not extrapolated to validate the remaining variables.

### 4.7. Eligible Inter-Epoch Comparisons

After the Stage 1 decisions reported in Section 4.5, only candidates that improve both internal indicators proceed to the inter-epoch continuity assessment. Table 6 separates the preliminary HDBSCAN-root-partition to BIRCH-Epoch-0 hand-off from the iterative BIRCH acceptance sequence. The value NMI = 0.768 is reported only to assess preservation of the initial density-supported structure and is not used as an acceptance threshold. The enrichment chain begins with accepted Epoch 0 versus candidate Epoch 1; subsequent candidates are compared with the latest accepted BIRCH epoch. Numerical hour of day has no NMI value because it failed Stage 1; consequently, Epoch 2 remained the reference for the subsequent categorical day-period candidate.

For the four eligible enrichment transitions, Epoch 1 establishes the initial BIRCH continuity value at NMI = 0.778 after passing Stage 1. Each subsequent accepted transition satisfies the non-decreasing rule relative to the most recent accepted transition: 0.787 ≥ 0.778 for Epoch 2, 0.847 ≥ 0.787 for Epoch 3 and 0.970 ≥ 0.847 for Epoch 4. Table 6 therefore documents the accepted chain from Epoch 0 to Epoch 4, while distinguishing the hour candidate rejected before Stage 2 from candidates that underwent inter-epoch comparison.

## 5. Discussion

The results support a controlled separation between discovery and characterisation through four empirical findings. First, HDBSCAN retains 1,763,591 of 5,897,113 tensor observations, defining the population to which all later interpretations apply. Secondly, the combined BIRCH–MiniBatchKMeans hand-off preserves substantial shared information with the HDBSCAN reference (NMI = 0.768) but produces different geometry, as shown by the lower Silhouette and higher Davies–Bouldin values. Thirdly, the candidate sequence demonstrates the operational effect of the two-stage rule: spatial variables, duration, categorical day period and route distance enter the accepted chain, whereas numerical hour is stopped before the continuity comparison. Finally, the runtime benchmark and per-representation profile quantify the computational rationale for repeated combined characterisation. The following subsections discuss these findings without treating the resulting partitions as semantic cluster explanations.

### 5.1. Impact of Noise Elimination

HDBSCAN labels 4,133,522 of the 5,897,113 tensor observations as noise and retains 1,763,591 observations, equivalent to 29.9% of the original tensor population. The fixed mask therefore removes 70.1% before enrichment. This decision has two measurable effects: every BIRCH epoch evaluates exactly the same 1,763,591 rows, and repeated processing is restricted to less than one third of the original observations. This improves inter-epoch comparability and tractability, but narrows the scope of inference to density-supported mobility. The excluded records cannot be described as erroneous or meaningless. Although the infrastructure supports several wireless interfaces, this case study uses only broadcast Wi-Fi detections. Consequently, Wi-Fi identifier rotation, intermittent Wi-Fi activity, variable radio range, scanning-window boundaries, local crowd density, communication delays and incomplete spatial coverage can produce fragmented or weakly supported transitions. Conversely, the same set may contain genuine rare trips that do not satisfy the density requirement of the initial partition. The 70.1% share must therefore be interpreted as an analytically excluded population generated jointly by Wi-Fi sensing conditions and low-density mobility, not as a measured error rate.

A descriptive analysis by origin sensor provides additional spatial context for the excluded population (Figure 7). Noise was distributed across the 136-location sensing network rather than dominated by a small number of sites: the largest individual sensor accounted for only 3.34% of all excluded observations, although sensor-specific exclusion rates varied substantially. Figure 7 displays the 20 sensors with the largest absolute excluded counts; within this subset, the proportion labelled as noise ranged from 71.15% to 81.72%. Sensor 16, which has the highest exclusion rate in this subset, is located in a high-footfall area of Malvarrosa beach. This contextual observation is compatible with a technological explanation in which denser device concentrations generate more fragmented pseudonymous traces because some mobile-device implementations repeatedly rotate randomised Wi-Fi MAC addresses over short periods. The resulting short-lived identifiers may have insufficient density support and therefore be assigned to noise. The location-specific evidence strengthens the plausibility of this mechanism, but does not establish it as the principal cause or demonstrate an exponential relationship, because crowd density, radio visibility, device mix, scanning conditions and other sensing effects were not independently measured.

### 5.2. Fixed Requested Resolution as an Experimental Control

The requested resolution remains fixed at *K* = 2176, while effective occupancy varies across the evaluated partitions. Table 6 reports 2176 occupied labels for the baseline and the first two accepted transitions, 2144 for the categorical day-period representation and 2160 for route distance, corresponding to utilisations of 100.000%, 98.529% and 99.265%. Because entropy is normalised against the invariant requested resolution, these occupancy changes are reported as structural diagnostics rather than used to redefine the comparison baseline.

This separation preserves a common denominator for inter-epoch comparison while making centroid use explicit. Occupancy remains a descriptive measure, whereas entropy and diversity remain the operational admissibility criteria. Accordingly, variation in occupied labels is considered when interpreting membership redistribution, but it does not alter the requested resolution or the acceptance decision.

### 5.3. Interpretation of the Enrichment Epochs

The enrichment results support two specific interpretations. The accepted chain shows progressive changes in membership distribution and continuity under a fixed population and requested resolution. The contrast between numerical hour and categorical day period shows that two representations of the same temporal concept can produce different admissibility outcomes.

Spatial coordinates increase relative entropy from 0.528 to 0.680 and Gini–Simpson diversity from 0.908 to 0.977, with NMI = 0.778 against the baseline. Journey duration raises the indicators to 0.686 and 0.978 and yields NMI = 0.787. Numerical hour then lowers them to 0.564 and 0.918, so the process stops before NMI is computed and Epoch 2 remains the reference. The categorical day-period reformulation increases the indicators to 0.899 and 0.998 and reaches NMI = 0.847 against Epoch 2; route distance subsequently raises them to 0.920 and 0.999 and reaches NMI = 0.970 against Epoch 3. These values demonstrate the exact effect of the gated procedure: Stage 1 determines whether comparison is eligible, and Stage 2 determines whether an eligible restructuring enters the accepted chain.

The temporal result should be interpreted as an encoding-specific outcome, not as evidence that temporal information is irrelevant. Numerical hour represents a cyclic phenomenon on a linear scale and fails the predefined distributional gate. The categorical alternative uses three mutually exclusive indicators and passes both stages. The experiment therefore supports the narrower conclusion that the categorical representation is more compatible with this framework and dataset under the stated rule. It does not establish whether the lower balance produced by numerical hour reflects poor encoding, genuine commuting peaks or both; profile-level analysis would be required to separate those explanations.

The temporal encoding in Section 4.1 and the acceptance outcomes reported here characterise the response of the partition to candidate representations; they do not provide temporal profiles or semantic labels for individual clusters. Such profiling is outside the present evaluation and remains part of the semantic-interpretation research line in the “Future Research” section.

Enrichment is also conditional on the accepted trajectory rather than an isolated assessment of each attribute. Each accepted variable changes the cumulative feature representation, the reference partition and, after the first eligible transition, the continuity value used for subsequent decisions. Earlier enrichments may therefore provide a context in which a later variable becomes admissible, suggesting possible complementarities between attributes. This is a methodological hypothesis, not an experimentally isolated order effect: the present study does not compare alternative candidate sequences under otherwise identical conditions. The preliminary shuffled control also differs in observation period and candidate order, so differences from the main experiment cannot be attributed to order alone. Random reassignment of attribute values and permutation of their introduction order address complementary questions and should be combined in a matched evaluation before explanatory complementarity is inferred.

### 5.4. Scalability and Methodological Implications

The computational contribution lies in the architecture that combines the distinct roles of HDBSCAN, BIRCH and MiniBatchKMeans. HDBSCAN performs one-off density discovery and noise identification. At each subsequent epoch, BIRCH compresses the fixed retained population into explicit CF-tree summaries, and MiniBatchKMeans performs the requested global partition over those summaries rather than over the complete observation matrix [4,5,6,13,14,15,16]. This hand-off allows the global stage to work on a targeted compressed representation while retaining counts and sufficient statistics for the original observations. The controlled benchmark in Section 4.3 and Table 3 measures the benefit of the complete procedure relative to HDBSCAN: at full scale, runtime falls from 281.322 to 74.005 min, corresponding to a 3.80× speed-up and an approximately 73.7% reduction. The standalone MiniBatchKMeans experiment is an ablation, not an equivalent replacement for the method: it confirms that direct centroid updating is inexpensive, but bypasses CF-tree construction, does not provide the same intermediate summaries and occupies only 58.180% of the requested centroids in the tensor-only control. The complementary profile in Section 4.4, Table 4 and Figure 6 shows how runtime, peak memory and CF-tree size vary as enriched representations accumulate. Because the benchmark and profile are separate experiments in a shared HPC environment, their absolute runtimes are not combined to estimate intrinsic CF-tree overhead. Each configuration was measured once, so run-to-run variability remains unquantified.

### 5.5. Limitations

- **Initial density-based discovery and excluded observations.** The framework depends on the initial HDBSCAN configuration and applies only to density-supported observations. The excluded population may combine acquisition artefacts, fragmented detections and meaningful rare or non-recurrent mobility. The present study provides a descriptive comparison by origin sensor, but does not identify semantically coherent groups within the excluded subset or determine whether individual observations correspond to sensing artefacts or genuine low-density mobility.
- **Wi-Fi sensing constraints.** The case study is based exclusively on broadcast Wi-Fi detections. Results are therefore conditioned by detector coverage, Wi-Fi activity, device discoverability, scanning and sensitivity settings, and successful communication and ingestion, while validated device-level error rates and a coverage map were unavailable.
- **Fixed resolution and acceptance rule.** Fixing *K* = 2176 ensures a common entropy reference and requested BIRCH resolution, but not identical occupancy or geometric equivalence with HDBSCAN. The strict Stage 1 rule and the non-decreasing NMI condition, *q_e_* ≥ *q_r_* for every eligible transition after Epoch 1, may reject genuine concentrated or strongly restructuring patterns, and candidate variables may be redundant.
- **Candidate-order dependence and shuffled-control scope.** Candidate admissibility is conditional on the previously accepted representation and continuity reference. Alternative introduction orders and attribute complementarities have not been evaluated under matched conditions. The preliminary control covers only two candidates in a different period and order; its five seeds vary random reassignment, not clustering initialisation or candidate order. It therefore does not establish real-sequence stability across clustering seeds, order robustness or attribute-specific semantic relevance. The observed increments and acceptance outcomes remain evidence for the reported unshuffled sequence under the operational rule, not for superiority over matched shuffled acceptance chains.
- **Performance evidence and generalisability.** Each performance configuration was profiled once, so run-to-run variability remains unmeasured and a complete matched repetition of every HDBSCAN enrichment stage in [3] remains unavailable. The standalone MiniBatchKMeans experiment is limited to the full-scale tensor baseline and functions as an ablation that bypasses the CF-tree; it neither reproduces the proposed compressed hand-off nor tests repeated enriched representations. The available benchmark therefore supports the runtime benefit of the complete BIRCH–MiniBatchKMeans procedure relative to HDBSCAN, but does not isolate the intrinsic cost of CF-tree construction or establish that fuller centroid occupancy is universally preferable. The method provides comparative evidence rather than semantic cluster descriptions, and its generalisability is limited to one city and one month.

Procedural reproducibility is supported by keeping the analytical population, row order, candidate sequence, preprocessing rules and clustering configuration unchanged across epochs. Section 4.1 reports the complete experimental specification; Section 4.3 and Section 4.4 and Table 3 and Table 4 document the execution environment and computational evidence. Together, these elements define the workflow and decision sequence needed to reproduce the study under the same data-access conditions. Empirical run-to-run stability remains untested because each performance configuration was profiled once.

## 6. Conclusions

This paper presents a noise-aware framework that extends the enrichment method in [3] by separating one-off HDBSCAN discovery from scalable characterisation over a fixed observation set. The characterisation stage combines BIRCH and MiniBatchKMeans: BIRCH constructs CF-tree summaries for each cumulative representation, and MiniBatchKMeans performs global partitioning over the resulting leaf summaries. The two-stage acceptance rule then distinguishes distributional admissibility from continuity with the latest accepted partition. These are controlled design choices for the Valencia case study rather than universal requirements for mobility clustering.

The experimental study demonstrates that the proposed architecture can be applied to a large-scale urban mobility dataset. From 5,897,113 tensor observations, HDBSCAN identified 4,133,522 noise observations and retained a stable set of 1,763,591 records. The combined BIRCH–MiniBatchKMeans procedure then evaluates the tensor baseline and the explanatory enrichments over the same population and a common requested resolution, while effective occupancy is reported separately.

The evidence supports three principal conclusions. First, noise elimination defines a stable analytical population and reduces repeated computation. The computational contribution then comes from the combined characterisation architecture: BIRCH compresses the retained observations into CF-tree leaf summaries, and MiniBatchKMeans performs the global partition over those summaries rather than over the complete matrix. Under the controlled full-scale benchmark, this procedure reduces runtime from 281.322 min for HDBSCAN to 74.005 min, corresponding to a 3.80× speed-up and an approximately 73.7% reduction. The standalone MiniBatchKMeans result is interpreted as an ablation showing that direct centroid updating is inexpensive, not as an equivalent substitute for the compressed repeated-enrichment workflow. The separate representation-level profile in Section 4.4 shows that the cost of the combined procedure varies with the accumulated features and CF-tree complexity; repeated-run variability remains unmeasured. Secondly, candidate-variable selection can be formalised through a fixed gated rule. Relative entropy and normalised Gini–Simpson diversity constitute the mandatory first stage; only when both increase does the process execute the consecutive-transition NMI comparison. For a candidate that fails internal validation, the NMI status is *Not computed*, because Stage 2 is not executed; only accepted partitions become references for later comparisons. The number of accepted enrichment epochs is not fixed in advance but emerges from the ordered candidate-variable set and the accept–reject decisions; the method can continue while unevaluated candidates remain. Applying the same staged rule makes acceptance, rejection, reformulation and continuation reproducible rather than dependent on post hoc interpretation. Because each condition is objectively computable, the complete decision cycle can be automated for other collections containing labels, clusters or predictions that require controlled partition comparison. Thirdly, the experiment maintains a common requested resolution across epochs while reporting effective occupancy separately.

The completed experiment reaches Epoch 4. The sequential decisions are as follows: acceptance of latitude and longitude in Epoch 1; acceptance of journey duration in Epoch 2; rejection of numerical hour of day against Epoch 2; acceptance of the reformulated categorical day-period variable as Epoch 3, again against Epoch 2; and acceptance of origin–destination route distance as Epoch 4 against Epoch 3. The distance variable represents the length of the journey required to travel between the two points through the transport network rather than Euclidean straight-line separation. This final sequence confirms that candidate variables can be rejected, reformulated and retested while preserving the rule that only accepted partitions become references for later comparisons.

### Future Research

Future work should test new ordered candidate sets, such as day type, weather, events, land use, public-transport accessibility and road-network characteristics—introducing variables individually and in alternative sequences. It should also examine alternative temporal and network representations while retaining the fixed accept–reject criterion.

A specific priority is to test whether earlier accepted variables facilitate the subsequent incorporation of complementary attributes. Because each acceptance updates the cumulative representation and partition reference, a variable may pass the two-stage rule in one introduction order but not another. This hypothesis should be evaluated by comparing alternative candidate sequences while holding the observation period, retained population, row order, encoding, preprocessing and clustering configuration fixed. Within each sequence, real candidates and shuffled counterparts should be evaluated against the same accepted reference, recording changes in distributional indicators, eligible NMI values and acceptance outcomes; complete shuffled trajectories should additionally be distinguished from these matched single-transition controls. Repeated shuffle seeds would assess sensitivity to random reassignment, while separate clustering seeds would assess algorithmic stability. This design would distinguish real-sequence enrichment, order sensitivity and possible attribute complementarity from improvements obtainable after reassignment, without treating distributional gains alone as semantic validation.

Robustness studies should evaluate alternative BIRCH resolutions and report occupancy, fragmentation, stability and computational cost. The performance analysis should be extended with repeated runs, standalone MiniBatchKMeans controls across enriched representations and a complete matched repeated-HDBSCAN enrichment condition. This research line will unify the runtime benchmark and representation-level profiling within a single evaluation protocol, with explicitly bounded and documented job allocations and application parallelism settings in the Slurm-managed HPC environment. A complementary research line is examining the excluded population through recursive HDBSCAN analyses to determine whether successive density levels reveal coherent low-density groups. This extension is not included in the present workflow because repeatedly reclustering millions of excluded observations has a high computational cost and, more importantly, requires a defensible stopping rule: without a convergence criterion, recursive extraction may continue to subdivide residual observations without establishing when the remaining structure is no longer analytically meaningful. Future work should therefore jointly bound computational complexity, minimum supported group size, stability across recursive levels and stopping conditions before integrating this analysis into an automated method. Further studies should compare final partition quality with that in [3], test alternative NMI bounds, candidate-order effects and variable redundancy, and examine the sensitivity of the valid-data mask to HDBSCAN parameters, samples and temporal windows.

Semantic interpretation should be strengthened by combining transition evidence with prototypes, rule extraction, local feature attribution or expert-defined urban categories, while evaluating fidelity, comprehensibility and transparency [12]. This would turn comparative stability results into actionable mobility descriptions without replacing the original unsupervised structure with an unvalidated surrogate.

Finally, the framework should be validated across cities, sensor layouts, periods and application domains. Future studies should quantify sensing and communication uncertainty, assess salt rotation as a privacy–utility trade-off, and test protected data settings. Incremental CF-tree updates, distributed processing, automated checks and machine-readable records could support longer enrichment sequences and continuous monitoring [13,14], moving the method towards a reusable platform for interpretable, privacy-aware machine learning.

## Figures and Tables

**Figure 1:**
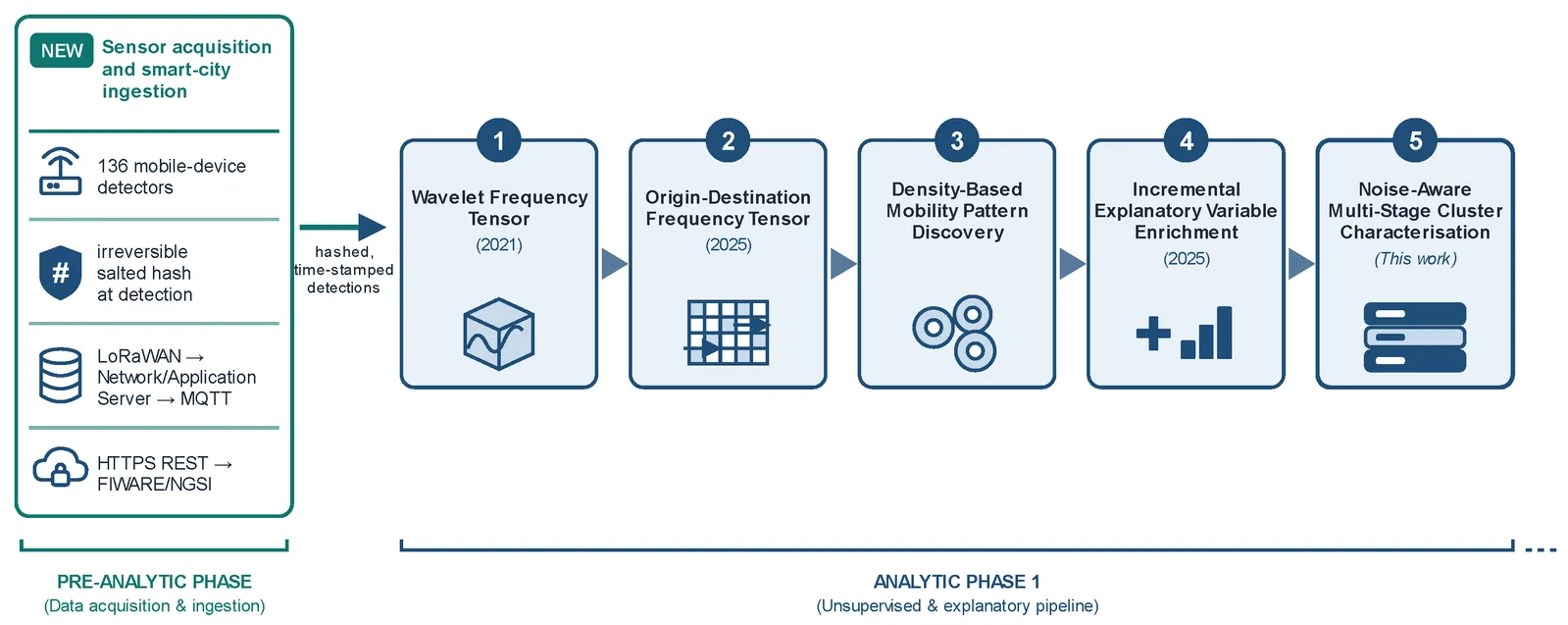
Sensor acquisition and smart-city ingestion linked to the development of the tensor-based mobility analysis framework, from the wavelet frequency tensor to the noise-aware cluster characterisation proposed in this study.

**Figure 2:**
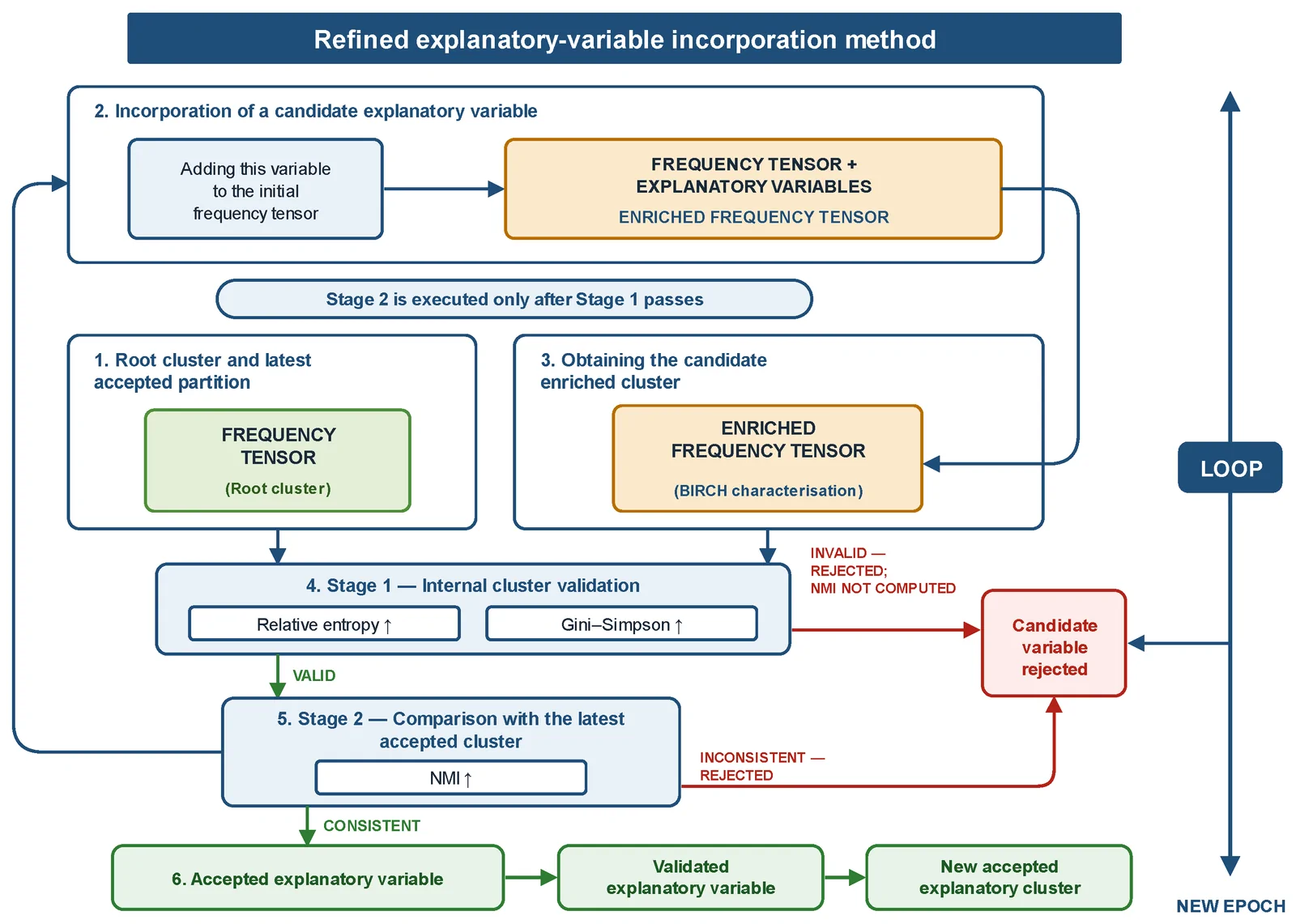
Two-stage evaluation of a candidate explanatory variable. Stage 1 requires increases in relative entropy and normalised Gini–Simpson diversity. Only candidates passing Stage 1 proceed to the NMI continuity assessment; rejected candidates leave the latest accepted representation unchanged.

**Figure 3:**
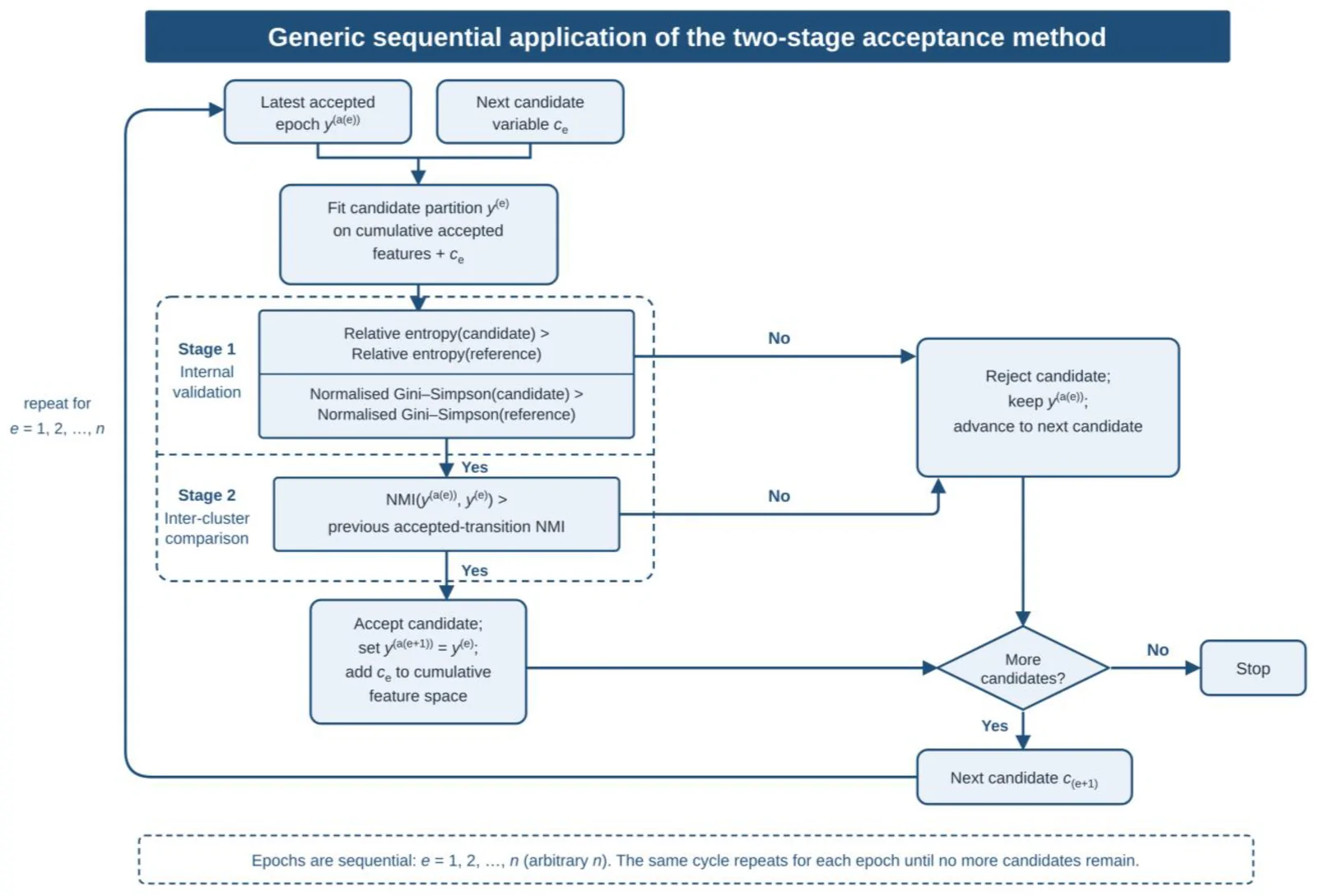
Sequential evaluation of an ordered set of candidate variables. Acceptance updates the cumulative feature space and reference partition; rejection preserves the latest accepted state. The first eligible transition establishes the NMI continuity reference, and subsequent eligible transitions must satisfy the non-decreasing condition defined in Section 3.6.

**Figure 4:**
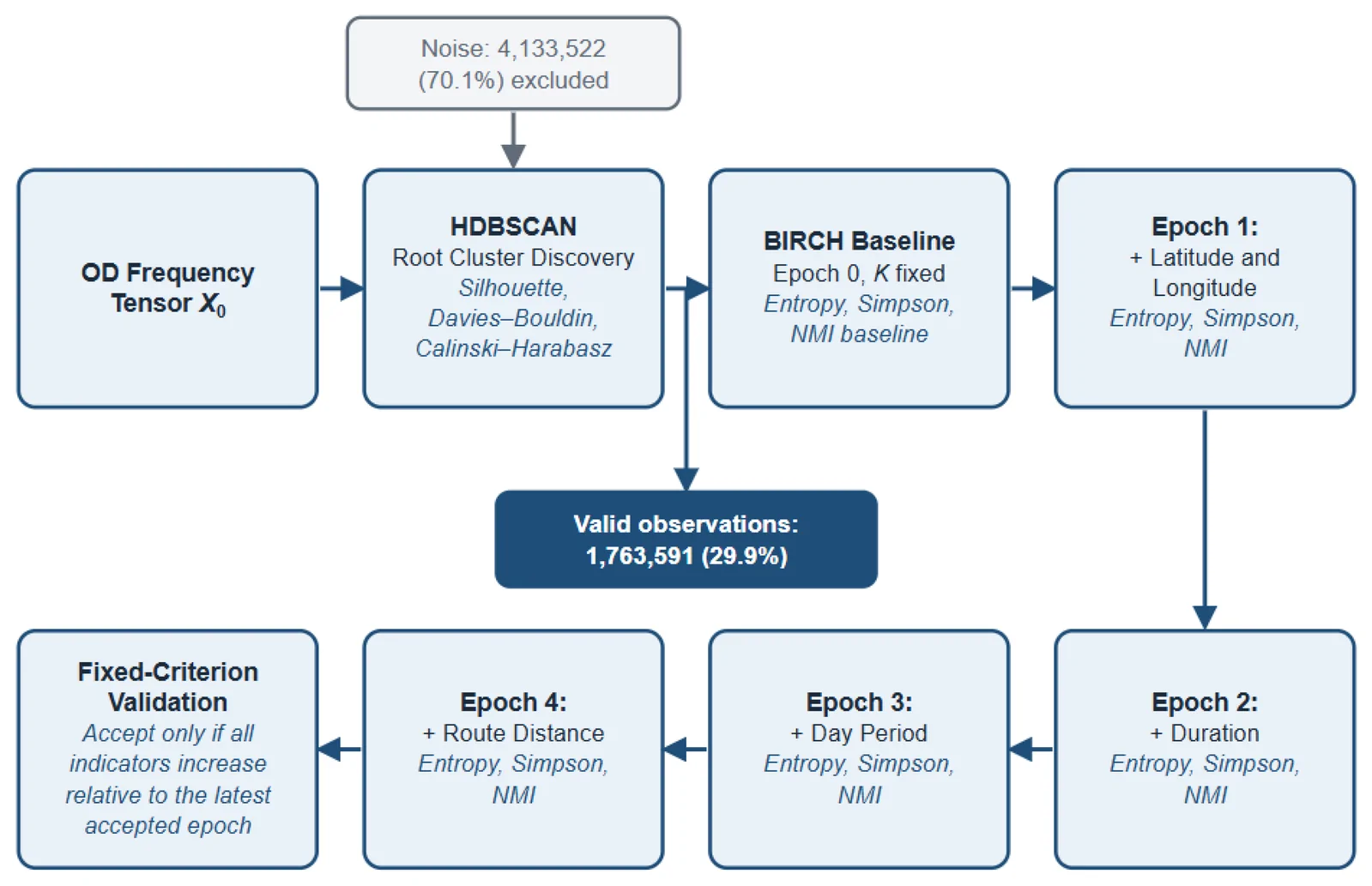
Valencia case-study pipeline: one-off HDBSCAN discovery excludes 4,133,522 observations (70.1%) and retains 1,763,591 (29.9%) for the tensor-only baseline and successive accepted enrichments with coordinates, journey duration, categorical day period and route distance. Candidate acceptance follows the two-stage rule in Section 3.6.

**Figure 5:**
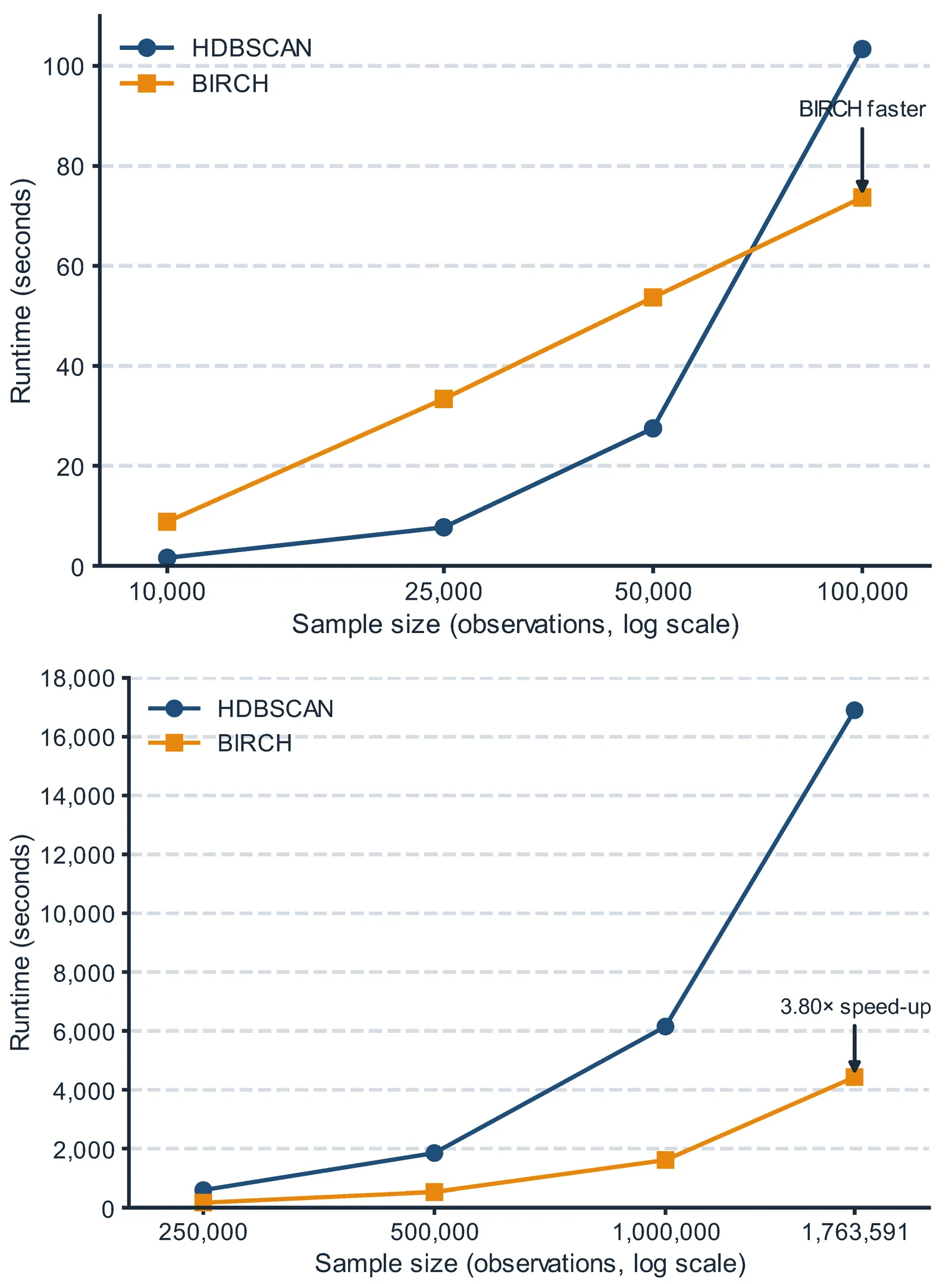
Runtime comparison between HDBSCAN and the complete BIRCH–MiniBatchKMeans procedure for samples of 250,000, 500,000, 1,000,000 and 1,763,591 observations. Values correspond to the single-run controlled benchmark in Table 3, not to the representation-level profile in Table 4.

**Figure 6:**
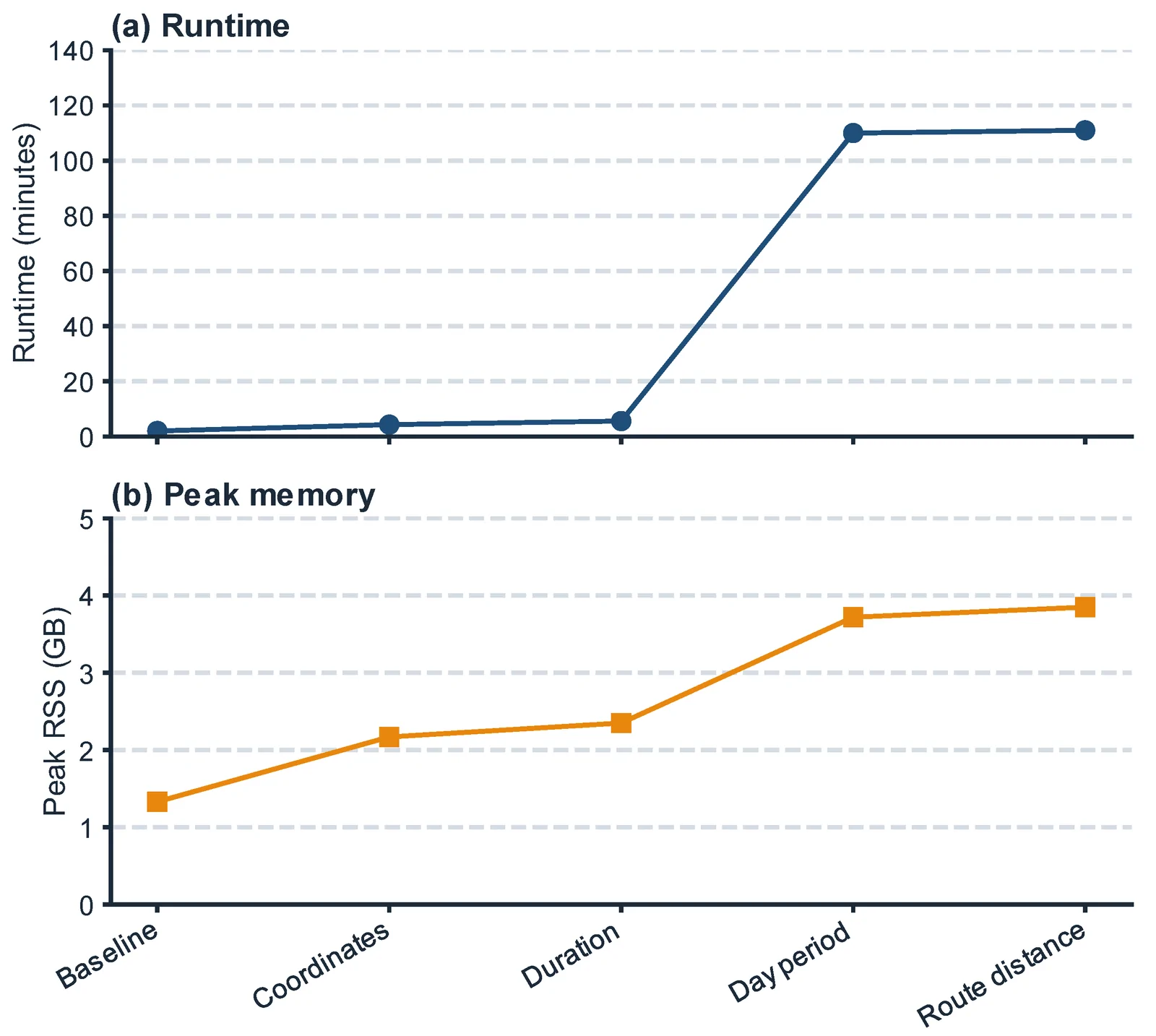
Runtime (min) and peak resident-set size (RSS, GB) for the tensor baseline and the cumulative coordinate, journey-duration, categorical day-period and route-distance representations, evaluated on the same 1,763,591 observations. Values correspond to Table 4 and are separate from the controlled benchmark in Table 3; the rejected numerical-hour candidate is not included.

**Figure 7:**
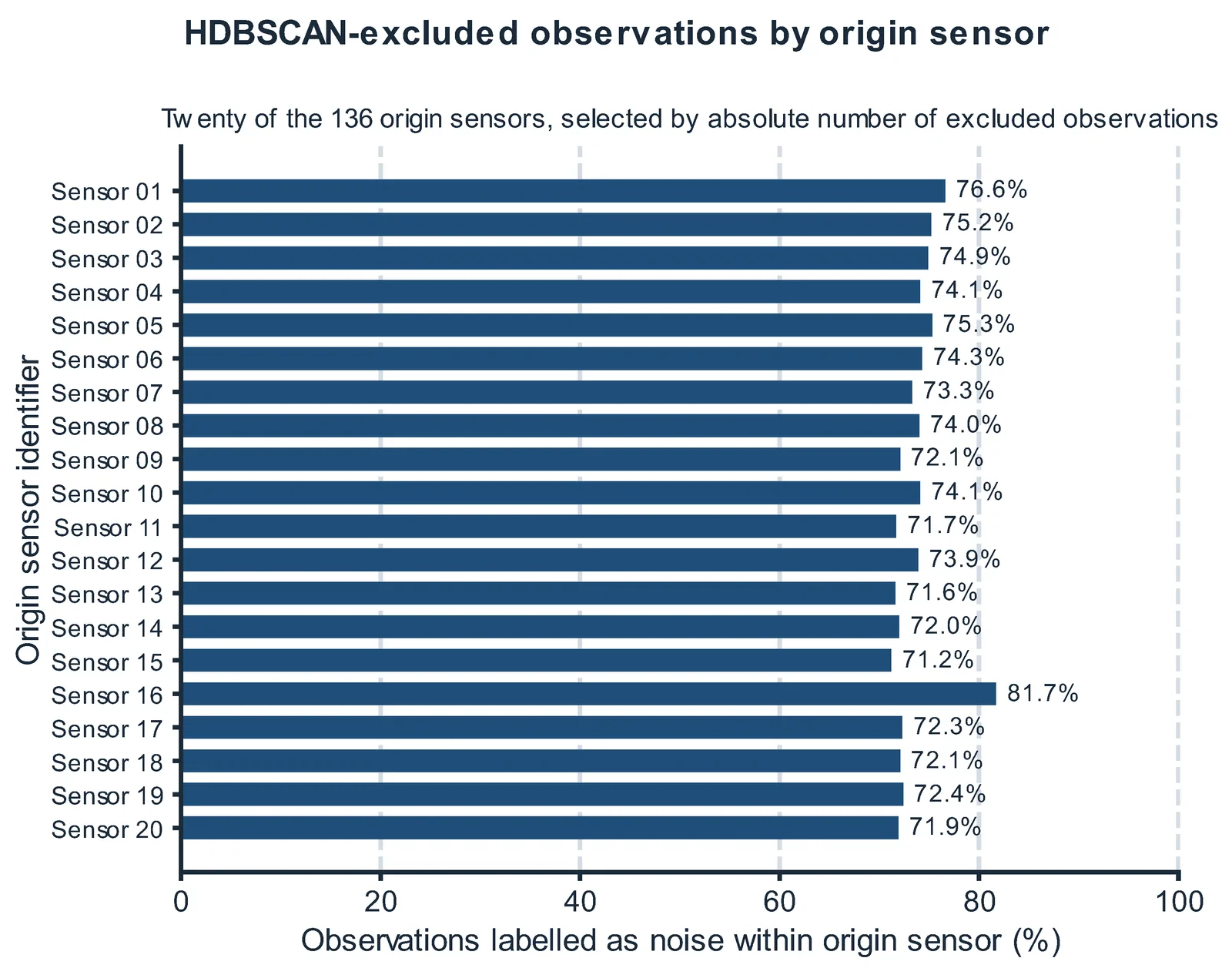
Percentage of observations labelled as noise by HDBSCAN at each of the 20 origin sensors with the largest absolute excluded counts, selected from the 136-location network and represented by anonymised identifiers. Percentages are calculated within each sensor, not as shares of the total excluded population. The noise label denotes density-based exclusion, not a measured sensing-error rate.

**Table 1:** Complementary roles of HDBSCAN and combined BIRCH–MiniBatchKMeans characterisation in the proposed framework.

Criterion	HDBSCAN	BIRCH–MiniBatchKMeans Characterisation
Role in the framework	One-off density discovery and noise identification	Repeated characterisation of candidate feature sets
Cluster-number requirement	No predefined number of clusters	Uses the reference requested resolution *K* in the global stage
Geometric behaviour	Supports irregular groups and heterogeneous density regions	Global centroid-based stage favours compact, variance-minimising partitions
Scalability mechanism	Density hierarchy is computationally demanding at large scale	CF-tree compresses observations into count, linear-sum and squared-sum summaries
Use across epochs	Executed once before enrichment	Executed repeatedly under a fixed configuration

**Table 2:** Comparison of the HDBSCAN reference partition and the combined BIRCH–MiniBatchKMeans tensor baseline. ARI and NMI compare the two partitions jointly.

Measure	HDBSCAN Reference	Combined Baseline	Interpretation
Observations	1,763,591	1,763,591	Identical retained population
Clusters/occupied centroids	2176	2176 requested/2176 occupied	Common requested resolution
Silhouette	0.835	0.276	Cohesion and separation
Davies–Bouldin	0.628	1.198	Compactness and separation
Calinski–Harabasz	24,187.035	6,920,681.897	Between-/within-cluster dispersion
Relative entropy	0.697	0.528	Membership distribution
Gini–Simpson diversity	0.964	0.908	Membership diversity
ARI	0.531		Pairwise agreement
NMI	0.768		Shared information

**Table 3:** Controlled runtime benchmark for HDBSCAN and the complete BIRCH–MiniBatchKMeans procedure across increasing sample sizes, with a full-scale standalone MiniBatchKMeans ablation.

Sample Size	Procedure	Runtime (s)	Runtime (min)	Speed-Up vs. HDBSCAN (×)	Clusters/Occupied Centroids	Utilisation/Noise (%)
250,000	HDBSCAN	548.355	9.139	1.00	234	Noise: 25.499
	BIRCH + MiniBatchKMeans	151.355	2.523	3.62	2176	Occupancy: 100.000
500,000	HDBSCAN	1795.112	29.919	1.00	428	Noise: 21.016
	BIRCH + MiniBatchKMeans	486.962	8.116	3.69	2175	Occupancy: 99.954
1,000,000	HDBSCAN	6161.043	102.684	1.00	842	Noise: 17.656
	BIRCH + MiniBatchKMeans	1567.666	26.128	3.93	2176	Occupancy: 100.000
1,763,591	HDBSCAN	16,879.325	281.322	1.00	1454	Noise: 15.186
	BIRCH + MiniBatchKMeans	4440.321	74.005	3.80	2176	Occupancy: 100.000
	Standalone MiniBatchKMeans	33.112	0.552	509.77	1266	Occupancy: 58.180

**Table 4:** Representation-level performance profile obtained during a complete reproduction of the combined BIRCH–MiniBatchKMeans enrichment procedure; these timings are not directly comparable with the controlled benchmark in Table 3.

Evaluated Representation	Features	Runtime (min)	Peak RSS (GB)	CF-Tree Leaf Nodes	Leaf Subclusters/Centres
Tensor baseline	15	1.983	1.334	3519	105,709
Tensor + latitude and longitude	19	4.020	2.151	16,795	501,136
Tensor + coordinates + journey duration	20	5.005	2.341	20,158	599,703
Categorical day-period representation	23	110.186	3.712	27,629	822,331
Route-distance representation	24	111.263	3.843	28,932	857,769

**Table 5:** Preliminary real and shuffled control results for the first two enrichment candidates. Shuffled values report the range across seeds 11, 22, 33, 44 and 55.

Epoch/Candidate	Variant	Relative Entropy	Gini–Simpson	Occupied Clusters	Occupancy (%)
Epoch 1—Latitude and longitude	Real	0.7651	0.9908	2176	100.000
	Shuffled range	0.7452–0.7697	0.9878–0.9917	2174–2175	99.908–99.954
Epoch 2—Journey duration	Real	0.7911	0.9935	2176	100.000
	Shuffled range	0.7779–0.8071	0.9903–0.9938	2174–2176	99.908–100.000

Note: The preliminary control was run on the first half of May 2025 with a different candidate order; its epoch labels and values are therefore not comparable with those in Section 4.5 and Table 6.

**Table 6:** Eligible NMI comparisons and acceptance outcomes through Epoch 4.

Comparison Type	Comparison	*K*_occ_	Utilisation (%)	NMI	Outcome
Initial baseline validation	HDBSCAN reference partition vs. BIRCH Epoch 0	2176	100.000	0.768	One-off validation; outside the acceptance chain
Acceptance transition 1	Accepted Epoch 0 vs. candidate Epoch 1	2176	100.000	0.778	Latitude and longitude accepted
Acceptance transition 2	Accepted Epoch 1 vs. candidate Epoch 2	2176	100.000	0.787	Journey duration accepted
Rejected candidate	Accepted Epoch 2 vs. numerical hour of day	2176	100.000	Not computed	Stage 1 failed; NMI not computed; Epoch 2 retained as reference
Acceptance transition 3	Accepted Epoch 2 vs. candidate Epoch 3	2144	98.529	0.847	Categorical day period accepted
Acceptance transition 4	Accepted Epoch 3 vs. candidate Epoch 4	2160	99.265	0.970	Route distance accepted

Table 2 reports the one-off HDBSCAN-reference-partition to Epoch 0 comparison in Section 4.2. Table 6 reports the sequential Stage 2 comparisons, the effective occupied cardinality (*K*_occ_) and centroid utilisation for every BIRCH epoch, and identifies numerical hour of day as *Not computed* because it failed Stage 1.

## Data Availability

The raw mobility detections cannot be made publicly available because they are subject to provider restrictions and privacy-preserving access conditions. Aggregated results and the code required to reproduce the tensor construction, clustering pipeline and validation procedure are available from the corresponding author upon reasonable request, subject to the applicable licence and data-protection constraints.
